# Persistence and Dose Escalation During Maintenance Phase and Use of Nonbiologic Medications Among Patients With Ulcerative Colitis Initiated on Ustekinumab in the United States

**DOI:** 10.1093/crocol/otad045

**Published:** 2023-09-04

**Authors:** Maryia Zhdanava, Ruizhi Zhao, Ameur M Manceur, Sumesh Kachroo, Patrick Lefebvre, Dominic Pilon

**Affiliations:** Analysis Group, Inc., Montreal, QC, Canada; Janssen Scientific Affairs, LLC, Horsham, PA, USA; Analysis Group, Inc., Montreal, QC, Canada; Janssen Scientific Affairs, LLC, Horsham, PA, USA; Analysis Group, Inc., Montreal, QC, Canada; Analysis Group, Inc., Montreal, QC, Canada

**Keywords:** corticosteroids, immunomodulators, biologics, real-world, insurance claims

## Abstract

**Background:**

Real-world data on treatment patterns among patients with ulcerative colitis (UC) initiated on ustekinumab are limited.

**Methods:**

Adults with UC initiated on ustekinumab (index date) between 10/18/2019 and 04/31/2022 were selected from a deidentified health insurance claims database (Symphony Health, an ICON plc Company, PatientSource). Persistence (no gaps in days of supply >120 days), persistence while being corticosteroid-free (no corticosteroid use for ≥14 days of supply after a 90-day grace period from index date) and dose escalation (≥2 consecutive subcutaneous claims ≥100% above daily maintenance dose) were described during the maintenance phase using Kaplan–Meier analysis. Nonbiologic treatments, among patients with ≥2 ustekinumab claims within 90 days post-index and ≥6 months of follow-up, were compared with logistic models 6 months post- versus pre-ustekinumab initiation.

**Results:**

6565 patients on ustekinumab entered the maintenance phase. At month 12 of the maintenance phase, 72.0% (95% confidence interval [CI]: 70.1%–73.9%) were persistent, 50.8% (95% CI: 48.7%–52.9%) were persistent and corticosteroid-free, and 19.2% (95% CI: 17.3%–21.3%) of patients had dose escalation. In the 6 months post- versus pre-ustekinumab initiation, the odds of nonbiologic medication use assessed in 4147 patients were significantly lower: 57% lower odds for corticosteroid, 46% for 60 cumulative days of corticosteroid, 42% for 5-aminosalicylic acid, and 24% for immunomodulators (all *P* < .001).

**Conclusions:**

Most patients with UC reaching the maintenance phase on ustekinumab remained persistent after 12 months of maintenance therapy. Nonbiologic medication use post-ustekinumab initiation was significantly lower, notably for corticosteroids. Given the multiple complications associated with chronic corticosteroid use, this reduction can be seen as clinically relevant and informs treatment choice for patients with UC.

## Introduction

Ulcerative colitis (UC) is a chronic inflammatory bowel disease of the colon and rectum.^1^ In 2014, there were an estimated 907 000 individuals with UC in the United States, and the incidence and prevalence of UC are increasing globally.^1,2^ UC is a progressive disease with a potentially debilitating course, and patients typically experience alternating episodes of active disease and periods of remission.^1,3^

Treatment goals include inducing and maintaining steroid-free remission, improving quality of life, promoting mucosal healing, and preventing hospitalization, surgery, and cancer.^3,4^ While there are no cures for UC, several treatment options are available for patients.^1,3,4^ Treatment algorithms depend on disease severity, and are based on a treat-to-target approach whereby the treatment is adjusted as needed according to the patient’s response in order to achieve and maintain remission.^5^ For patients with mild-to-moderate UC, medical treatment options begin with 5-aminosalicylic acid (5-ASA) and corticosteroids.^3,4^ For patients who progress to or present with moderate-to-severe UC, medical treatment options are corticosteroids, immunomodulators, or advanced therapies, including biologics and small molecules.^1,4,6^ Additionally, combination therapy with biologic and other agents (eg, corticosteroids, immunomodulators) is common; however, it may increase the risk of serious infection compared to monotherapy.^7^ Some patients may eventually require surgical intervention if severe complications arise or the disease is unable to be controlled with medical therapy.^1^

Recently, second-generation biologics providing the targeted inhibition of multiple interleukins (IL) have further expanded the treatment landscape. Ustekinumab, an anti-interleukin 12 (IL-12) and anti-interleukin 23 (IL-23) agent, was approved by the US Food and Drug Administration in October 2019 as a treatment for moderate-to-severe UC based on the results of the phase 3 UNIFI trial (260–520 mg intravenous induction and 90 mg every 8 weeks subcutaneous maintenance).^8,9^ Given the short time since its approval, there is limited real-world information on ustekinumab treatment patterns in patients with UC in the United States,^10^ especially with respect to persistence and dose escalation,^11^ both useful measures of medication performance in the real world.^12–15^ As treatment response varies according to the treatment received,^16^ data regarding ustekinumab performance can guide therapeutic decision-making in clinical practice.

This study aimed to evaluate treatment patterns among patients with UC initiated on ustekinumab in a real-world setting. Specifically, we aimed to describe persistence and dose escalation during the maintenance phase among patients with UC initiated on ustekinumab and to compare nonbiologic medication use pre- and post-ustekinumab initiation among patients with UC.

## Methods

### Data Source

The Symphony Health (SHS database), an ICON plc Company, PatientSource (04/01/2017–03/31/2022) database was used. The SHS database is an open claims longitudinal patient data source, which captures prescription claims containing data on medical utilization and costs across the United States from commercial and government (Medicare and Medicaid) sources. The open claims nature means that individual patient healthcare activity is captured regardless of healthcare plan changes/switching if the patient uses providers from the network that supplies data to the database. The SHS database complies with the patient confidentiality requirements of the Health Insurance Portability and Accountability Act and is deidentified. Therefore, no institutional review board approval was necessary.

### Study Design

A retrospective cohort design was used. Patients with UC (International Classification of Diseases, Ninth Revision [ICD-9]: 556.x; International Classification of Diseases, 10th Revision [ICD-10]: K51.x) initiated on ustekinumab were selected. The intake period spanned from 10/18/2019 (the date of ustekinumab approval for UC in the United States^8^) to 03/31/2022 (end of data availability at study time). The index date corresponded to the date of ustekinumab initiation (first recorded claim for ustekinumab use in pharmacy or medical file, if the first claim occurred during the intake period).

Two populations of patients with UC initiated on ustekinumab were selected from the overall population of patients initiating ustekinumab. Population 1 was used for the analyses of persistence and dose escalation among patients who had reached maintenance. Population 2 was used for the analysis of nonbiologic medication use before and after ustekinumab initiation (henceforth pre- and post-ustekinumab initiation periods), and was selected to ensure that medication use could be observed over symmetric periods of time, and that patients were exposed to ustekinumab in the post-ustekinumab initiation period. Populations 1 and 2 were independent subsets of the overall population, and a patient could be included in either or both of the populations, as described in “Sample selection.”

For the persistence and dose escalation analyses, the baseline period to describe patients before the initiation of ustekinumab corresponded to the 12 months before the index date, and the follow-up period spanned from the index date to the earliest of the end of the data availability, or the last indicator of clinical activity (ie, any medical, pharmacy, or procedure claim in the database). Given the open nature of the SHS database, the first and last diagnosis, procedure, or pharmacy claim were used as the start and end dates of the clinical activity period.

For the nonbiologic medication use pre-/post-ustekinumab initiation analysis, the baseline and follow-up periods were of the same length, 6 months before and after the index date.

### Sample Selection

**Figure 1.**
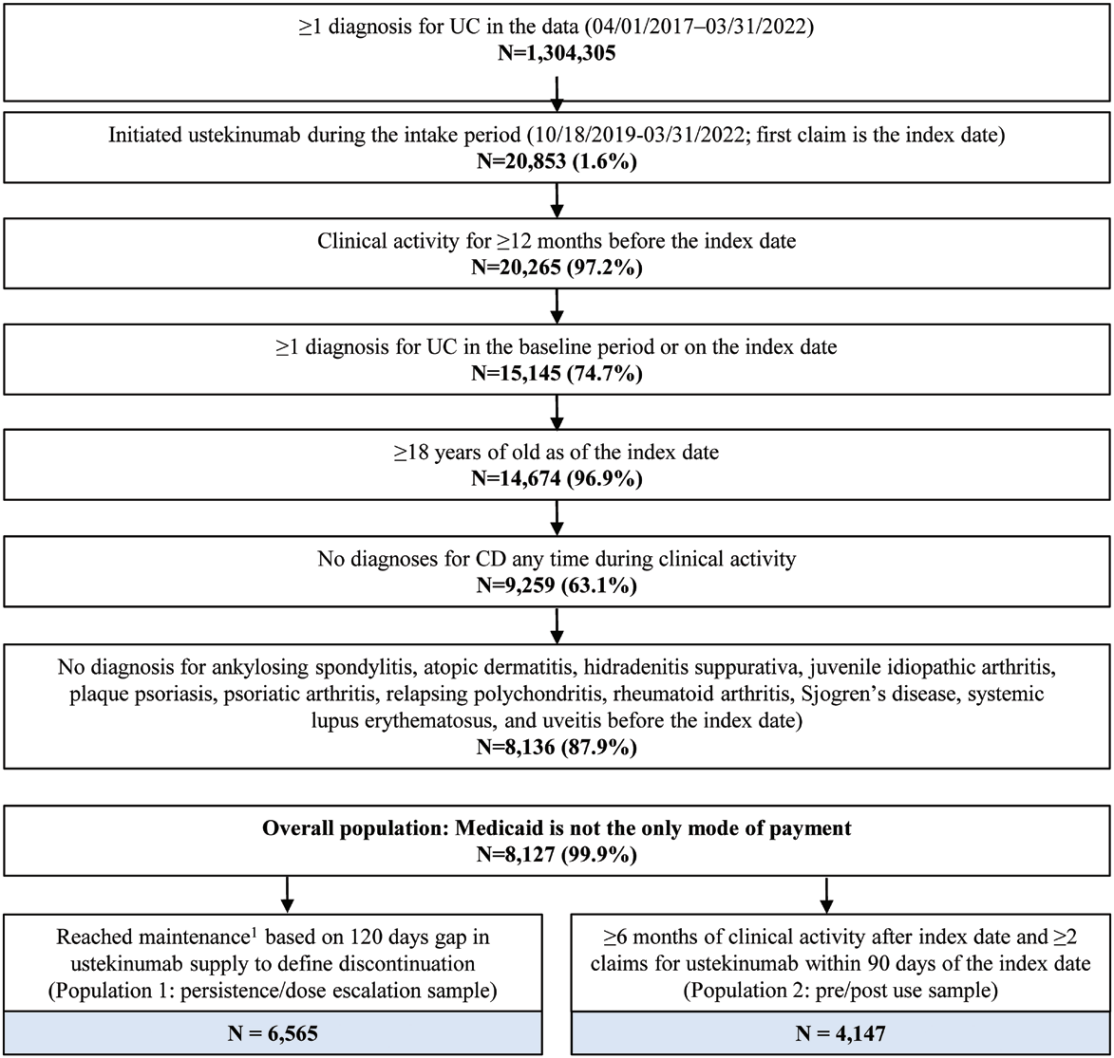
Sample selection flowchart. ^1^The start of the maintenance phase was defined as the first subcutaneous claim for ustekinumab. Abbreviations: CD: Crohn’s disease; UC: ulcerative colitis.

Patients included in the overall population were required to have ≥1 claim for ustekinumab during the intake period, have ≥12 months of clinical activity before the index date (initiation of ustekinumab), be ≥18 years old as of index date, have ≥1 diagnosis for UC in the baseline period before or on the index date, and have private insurance (Figure 1). Patients were excluded from the study population if they had ≥1 claim for Crohn’s disease (ICD-9: 550.x; ICD-10: K50.x) before or after the index date, ≥1 claim for ankylosing spondylitis, atopic dermatitis, hidradenitis suppurativa, juvenile idiopathic arthritis, plaque psoriasis, psoriatic arthritis, relapsing polychondritis, rheumatoid arthritis, Sjogren’s disease, systemic lupus erythematosus, and uveitis before the index date, and if their only method of payment was Medicaid during the period of clinical activity. The exclusion of other immune-mediated diseases was required to ensure that ustekinumab was used for UC.

Patients in population 1, in addition to the overall population criteria, were required to have reached maintenance. Patients in population 2, in addition to the overall population criteria, were required to have ≥6 months of clinical activity after the index date and ≥2 claims for ustekinumab within 90 days of the index date.

### Outcome Measures

#### Imputation of days of supply

Measures of treatment patterns in claims data utilize information on claim dates and days of supply (ie, the number of days the prescription fill is supposed to last). Days of supply in pharmacy claims for nonoral medications like ustekinumab may be inconsistent with the frequency of administration per label; in medical claims (for instance, intravenous ustekinumab), days of supply are not available. This motivates the need for the imputation of days of supply.

Imputation of the days of supply for ustekinumab was based on the US label frequency of administration, the mode of days of supply, and time to the next claim observed in the data.^8^ For pharmacy claims with days of supply <34 days, or for medical and pharmacy claims with missing days of supply, days of supply were imputed based on the duration of the gap until the next claim: 28 days if the gap was <38 days, 42 days if the gap was 38 to 46 days, and 56 days if the gap was >46 days. If there was no next claim, days of supply of the previous claim were carried forward, or the per-label number of days of supply (56 days) was imputed. The imputation of values of days of supply for ustekinumab intended to preserve the observed variation in these values in the data, which could be due to variations in dosing schedules in clinical practice.^17^ This imputation method was used in preceding research.^18,19^

#### Persistence during maintenance phase

Persistence on ustekinumab was measured from the first maintenance claim (ie, first subcutaneous claim) among patients persistent on ustekinumab between the index date and the start of the maintenance phase. Persistence on ustekinumab was defined as no gap in consecutive days of ustekinumab supply of over twice the labeled dosing interval^8^ (>120 days) in the primary analysis, and over 1.5 times the labeled dosing interval (>90 days) in the sensitivity analysis (ie, using a more conservative definition of discontinuation with a shorter gap).

In addition, the composite outcome of persistence (based on primary gap definition) while being corticosteroid-free was reported among patients who were corticosteroid-free from the index date to the start of the maintenance phase. Being corticosteroid-free was defined as no corticosteroid use for ≥14 days of supply after a 90-day grace period from the index date to allow for corticosteroid tapering following biologic initiation.

Persistent time was measured from the start of the maintenance phase until the discontinuation date (ie, the last day of ustekinumab supply before the exposure gap). For the composite outcome of persistence while corticosteroid-free, persistent time was measured from the start of the maintenance phase until the earliest of the discontinuation date or corticosteroid-use event date. Patients without discontinuation or corticosteroid use were censored on the last day of ustekinumab supply during the follow-up period. Censored patients did not have an event during the available follow-up time.

#### Dose escalation during maintenance phase

In claims data, dose escalation could be identified as a higher dose in milligrams in a given ustekinumab claim or a higher frequency of administration while the dose in milligrams remains the same. Each claim of ustekinumab typically provides a fill date, the total dose (in mg), and the number of days of supply.

Dose escalation was analyzed among patients persistent on ustekinumab between the index date and the start of maintenance phase (primary definition, >120 days gap to define discontinuation). The daily dose (exposure) at each maintenance claim was calculated as the total dose divided by the days of supply. Dose escalation was defined as≥2 consecutive subcutaneous claims ≥100% above the daily maintenance dose of ustekinumab per label (1.6 mg/day)^8^; the date of the first of 2 claims was the date of the dose escalation. Patients without dose escalation were censored at the discontinuation date or the last day of ustekinumab supply during the follow-up period, whichever occurred first. Given the focus on the maintenance phase, only subcutaneous claims were included in the dose escalation analysis and intravenous administration (ie, re-induction) was not taken into account.

#### Use of nonbiologic medication pre- and post-ustekinumab initiation

Use of nonbiologic medication (ie, recorded claim of medication use in the pharmacy of medical file) was reported in the 6-month period pre- and post-ustekinumab initiation among patients with (1) ≥6 months of clinical activity after the index date, and (2) ≥2 claims for ustekinumab within 90 days of the index date. This was done to ensure that medication use could be observed and that patients were exposed to ustekinumab in the post-period.

#### Statistical analysis

Descriptive statistics were reported as mean with standard deviation (SD) for continuous variables and as frequencies with proportion for categorical variables. Persistence and dose escalation were assessed using Kaplan–Meier analyses to account for censoring, and rates with the 95% confidence intervals (CIs) were reported. Nonbiologic medication use pre- and post-ustekinumab initiation was compared with generalized estimating equation models with a binomial distribution and a logit link adjusted for repeated measurements on the same patient. Odds ratios (ORs) comparing use post- and pre-ustekinumab initiation and 95% CIs were reported.

## Results

### Study Populations

A total of 8127 patients with UC initiated on ustekinumab were selected in the overall population of patients initiating ustekinumab, among whom 6565 patients entered the maintenance phase on ustekinumab and were included in the persistence and dose escalation analysis (population 1). A total of 4147 patients met the criteria for the pre/post-use of nonbiologic medications analysis (population 2; Figure 1).

### Persistence and Dose Escalation During the Maintenance Phase (Population 1)

Among the 6565 patients who persisted on ustekinumab to the maintenance phase, the mean age was 43.8 years, and 48.0% were female. The majority had at least 1 claim of corticosteroids at baseline (69.7%) and used biologic or advanced therapy prior to the index date (51.6%; Table 1).

**Table 1.** Baseline characteristics among patients with UC initiated on ustekinumab.^a^

Mean ± SD or *n* (%)	Population 1: Persistence/dose escalation sample *N* = 6565	Population 2: Pre/post-use sample *N* = 4147
Age	43.8 ± 15.6	44.0 ± 15.6
Female	3153 (48.0)	1987 (47.9)
Year of index date		
2019	195 (3.0%)	162 (3.9%)
2020	2319 (35.3%)	2057 (49.6%)
2021	3254 (49.6%)	1928 (46.5%)
2022	797 (12.1%)	0 (0.0%)
Payer		
Commercial insurance	5,534 (84.3%)	3,509 (84.6%)
Medicare	530 (8.1%)	338 (8.2%)
Medicaid	389 (5.9%)	229 (5.5%)
Other	112 (1.7%)	71 (1.7%)
Quan–Charlson Comorbidity Index^b^	0.28 ± 0.90	0.20 ± 0.70
Symptoms and comorbidities (5 most frequent)		
Diarrhea	1475 (22.5)	731 (17.6)
Inflammatory arthritis or enteropathic arthropathies	1355 (20.6)	694 (16.7)
Pain	1210 (18.4)	532 (12.8)
Anemia	1168 (17.8)	571 (13.8)
Cardiovascular disease	1040 (15.8)	505 (12.2)
Medications		
Corticosteroids	4575 (69.7)	2565 (61.9)
Continuous use ≥60 days^c^	2322 (35.4)	1097 (26.5)
Continuous use ≥90 days^c^	1413 (21.5)	576 (13.9)
Cumulative use ≥60 days^c^	2948 (44.9)	1346 (32.5)
Cumulative use ≥90 days^c^	2153 (32.8)	775 (18.7)
Biologics and advanced therapy	3386 (51.6)	2000 (48.2)
Tumor Necrosis Factor inhibitors	2133 (32.5)	1163 (28.0)
Anti-integrin agent (vedolizumab)	1139 (17.3)	603 (14.5)
Janus kinase inhibitors (tofacitinib)	581 (8.8)	350 (8.4)
Number of biologics/advanced therapy agents	0.60 ± 0.60	0.51 ± 0.60
Use of 2 or more	500 (7.6)	124 (3.0)
Use of 3 or more	47 (0.7)	4 (0.1)
Conventional therapy	3750 (57.1)	1998 (48.2)
5-ASA	3226 (49.1)	1655 (39.9)
Immunomodulators	1160 (17.7)	605 (14.6)
Opioids	1651 (25.1)	729 (17.6)
Gastrointestinal antispasmodics	1043 (15.9)	441 (10.6)
Antibiotics	960 (14.6)	366 (8.8)
Antidiarrheal	405 (6.2)	194 (4.7)

Abbreviations: 5-ASA, 5-aminosalicylic acid; SD, standard deviation; UC, ulcerative colitis.

^a^For the persistence analysis, the baseline characteristics were reported during the 12-month periodbefore initiation of ustekinumab. For the pre/post-analysis, the baseline characteristics were reported during the 6-month period before initiation of ustekinumab.

^b^Quan H, Sundararajan V, Halfon P, et al. Medical Care. 2005;43(11):1130–1139..

^c^Cumulative and nonoverlapping days of supply over the baseline period were included. A gap of 14 days of supply was used to define continuous episode of use. Cumulative use was defined with nonoverlapping days of supply.

**Figure 2.**
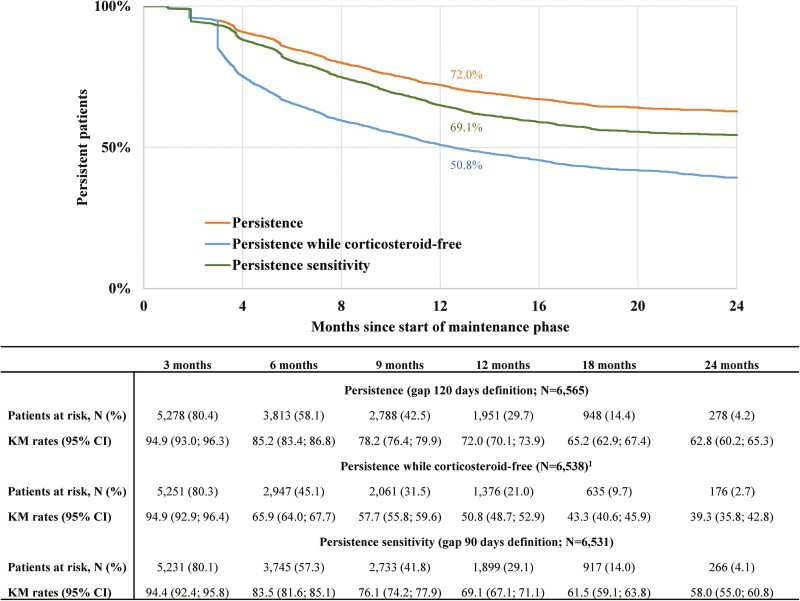
Persistent time on ustekinumab during maintenance phase among patients with UC (population 1). Persistent time was measured from the first maintenance claim until the discontinuation date or, for the composite outcome, until the earliest among the discontinuation date or corticosteroid use date. Patients without discontinuation or corticosteroid use were censored at the earliest of the last day of index biologic supply during the follow-up period. ^1^Patients were required to be persistent and corticosteroid-free at the maintenance phase start. Abbreviations: CI: confidence interval; UC: ulcerative colitis.

The mean follow-up from the maintenance phase start to the end of clinical activity was 11.3 months (SD: 7.8 months) with a median of 10.3 months. At 12 months after the start of the maintenance phase, 72.0% (95% CI: 70.1%–73.9%) of patients were persistent on ustekinumab given a 120-day gap definition (Figure 2). Among the 6538 patients who had reached the maintenance phase while persistent and corticosteroid-free, the rate of being persistent and corticosteroid-free at 12 months was 50.8% (95% CI: 48.7%–52.9%; Figure 2). In the sensitivity analysis with >90-day gap to define discontinuation, among the 6531 patients who had reached maintenance phase while persistent based on that gap, 69.1% (95% CI: 67.1%–71.1%) of patients were persistent at 12 months (Figure 2).

**Figure 3.**
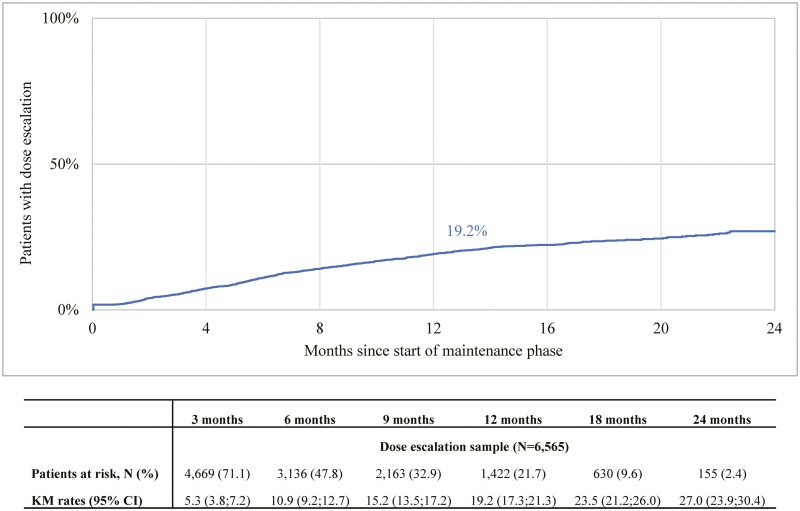
Time to dose escalation of ustekinumab (≥100% increase above US-labeled dose for 2 consecutive subcutaneous administrations) during the maintenance phase among patients with UC (population 1). Time to dose escalation was measured from the first maintenance claim until the first dose escalation event. Patients without dose escalation were censored at the earliest of the discontinuation date or the last day of index biologic supply during the follow-up period. Abbreviations: CI, confidence interval; UC, ulcerative colitis.

At 12 months after the start of the maintenance phase, 19.2% (95% CI: 17.3%–21.3%) of patients had dose escalation ≥100% above the US-labeled dose for at least 2 consecutive subcutaneous claims (Figure 3).

### Nonbiologic Medication Use Pre- and Post-Ustekinumab Initiation (Population 2)

Among the 4147 patients included in the nonbiologic medication use pre- and post-ustekinumab initiation analysis, the mean age was 44.0 years, and 47.9% were female. Patients had fewer comorbidities and lower medication use compared to population 1, as expected since the baseline duration was 6 months compared to 12 months in population 1 (Table 1).

**Figure 4.**
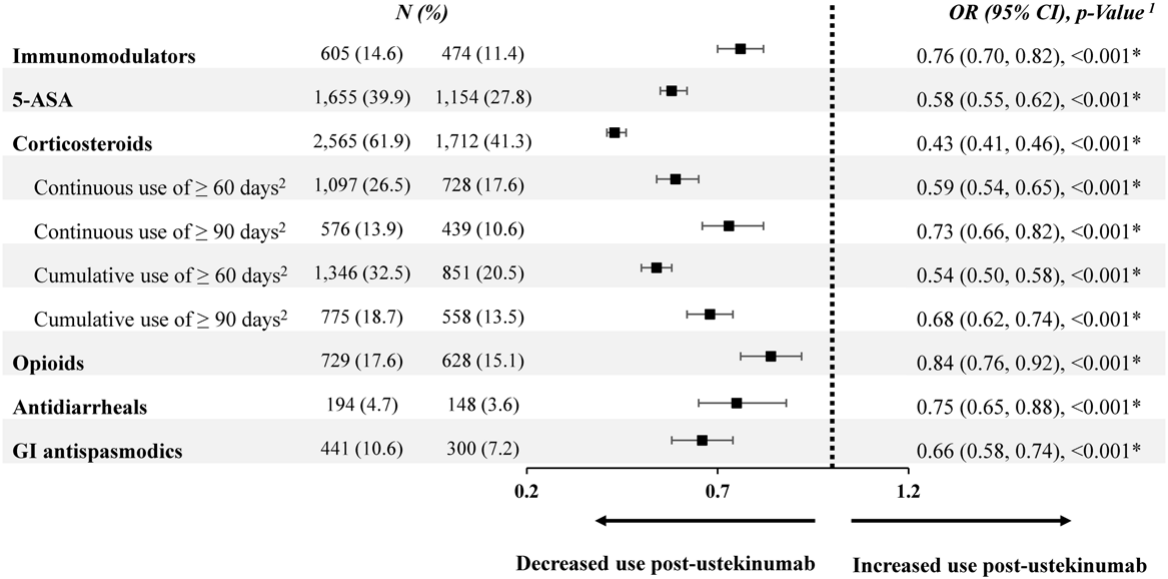
Nonbiologic medication use 6 months post- versus pre-ustekinumab initiation among patients with UC (N = 4,147, population 2). ^1^Obtained from a logistic regression model estimated by generalized estimating equation adjusting for repeated measures per patient. *P-value ≤ .05. ^2^For continuous use of corticosteroids, a gap of 14 days of supply was tolerated (ie, the episode of use continued even when there were no days of supply of corticosteroids for 14 consecutive days). For cumulative use of corticosteroids, nonoverlapping days of supply were summed. Abbreviations: 5-ASA, 5-aminosalicylic acid; CI, confidence interval; GI, gastrointestinal; OR, odds ratio.

In the 6 months post-ustekinumab initiation, the odds of nonbiologic medication use were significantly lowered by 57% for any corticosteroid use, 46% for 60 cumulative days of corticosteroid use, 42% for 5-ASA, and 24% for immunomodulators (all *P* < .001). Odds of opioids, antidiarrheals, and gastrointestinal antispasmodics use were also significantly lower in the 6 months post-ustekinumab initiation compared to the 6 months pre initiation (Figure 4).

In particular, for corticosteroids (ie, budesonide, dexamethasone, hydrocortisone, prednisolone, prednisone), there were 1479 (35.7%) patients who were continuous users (in the pre- and post-period) and 233 (5.6%) who were new users. Among the continuous users, the first corticosteroid prescription occurred on average 42 days after the initiation of ustekinumab, whereas the first prescription occurred 81 days after initiation among the new steroid users.

New starts of other nonbiologic agents also represented a small proportion of the total population under study (ie, 3.2% for immunomodulators, 4.0% for 5-ASA, 7.8% for opioids, 1.4% for antidiarrheals, and 2.9 for GI antispasmodics).

## Discussion

In this US real-world study of treatment patterns among patients with UC initiated on ustekinumab, we found that most patients reaching the maintenance phase remained persistent after 12 months of maintenance therapy. This estimate remained robust when a relatively more conservative definition of discontinuation was used to define persistence. Dose escalation, which has been used in clinical practice to achieve and maintain remission,^20,21^ was observed in approximately one-fifth of patients after 12 months of maintenance therapy. Finally, we found that nonbiologic medication use post-ustekinumab initiation, and notably corticosteroid use, was significantly lower in the 6 months post- versus pre-ustekinumab initiation.

Ustekinumab was approved for the treatment of moderate-to-severe UC in the United States in 2019.^8^ This approval was based on a randomized, double-blind, placebo-controlled clinical trial that assessed the efficacy and safety of ustekinumab in patients with moderate-to-severe UC.^8,9^ Given this relatively recent approval, there are few real-world studies examining real-world treatment patterns and the use of ustekinumab for patients with UC in clinical practice. Recently published studies did not compare rates of nonbiologic medication use before and after initiating ustekinumab,^11^ were limited to Europe, and evaluated relatively small populations of patients (ranging from 19 to 133) with active UC.^22–25^ Nonetheless, these studies provided evidence for the effectiveness of ustekinumab, with clinical remission rates of up to 53% after 1 year.^23^ They also reported high rates of treatment persistence (67% over a median follow-up of 32 weeks^25^ and 59% after 72 weeks^24^). To our knowledge, the present study is among the first to examine the treatment patterns of ustekinumab in a large population of patients with UC in the real world in the United States.

In the real-world setting, medication persistence or drug survival is a measure of clinical outcomes, such as effectiveness and safety, as well as other factors related to medication use, such as convenience and patients’ perception of risks/benefits associated with the medication.^12,14,26,27^ In this study, over 70% of patients were persistent to ustekinumab at 12 months, suggesting that some level of disease control was achieved for a substantial proportion of patients with UC reaching maintenance in the studied population. Persistence on a biologic may present additional advantages to the health system and to patients, as restart and switch can be interpreted as indicators of suboptimal treatment, which were found to be associated with higher healthcare costs^28^ and reduced quality of life^25^ among patients with IBD. The latter is an especially important consideration, given that in addition to improving clinical outcomes, a major goal of the treat-to-target strategy for IBD is to restore patients’ quality of life.^29^

Corticosteroids are efficacious in inducing remission, but guidelines recommend against their use for the maintenance of remission.^4^ In this study, over half of patients were persistent while being corticosteroid-free after 12 months of maintenance therapy and the pre-/post-analysis showed that 6 months after ustekinumab initiation, the odds of treatment with corticosteroids were 57% lower. Both patients and physicians have identified achieving and maintaining corticosteroid-free remission as a key goal for treating UC,^4,17,30,31^ as short- and long-term treatment with corticosteroids in patients with IBD may be associated with adverse effects including infection, hypertension, new-onset diabetes mellitus, osteoporosis, cataracts, and glaucoma,^32,33^ as well as an increased risk of death compared with alternative therapies.^34,35^ Additionally, corticosteroids do not promote mucosal healing—a therapeutic endpoint and treatment goal for UC^36^ that is associated with better outcomes such as clinical remission and avoidance of colectomy^37^—over the long term.^38^ While combination therapy might help some patients to achieve remission, reducing immunosuppression is also desirable as it decreases the risks of opportunistic infections and other complications.^7,39^ Furthermore, the results showed a reduction in the use of immunomodulators in the 6-month period after ustekinumab initiation. This pattern may be explained by clinical practice; indeed, a recent meta-analysis concluded that concomitant use of immunomodulators with ustekinumab did not present a benefit compared to ustekinumab monotherapy^40^ while the combination of biologics with immunomodulators was associated with increased toxicity compared to monotherapy.^41^

In real-world clinical practice, dose escalation has been used as a treatment option for patients who do not achieve optimal clinical response to induction therapy.^14^ In our study, approximately one-fifth of patients had dose escalation ≥100% above the US label-indicated daily maintenance dose of ustekinumab (for instance, an increase in the frequency of administration from every 8 weeks to every 4 weeks) at 12 months after the start of the maintenance phase. Recent real-world studies of dose intensification every 4 or 6 weeks in patients with UC have demonstrated the potential effectiveness of this treatment approach.^20,21^ Our results are also consistent with previous studies of patients with IBD or UC, which reported that between approximately one-fifth and one-third of patients underwent dose escalation in real-world clinical practice.^42,43^ Dose escalation has also been shown to be an effective treatment strategy in real-world studies of patients with Crohn’s disease.^17,44^

This study should be interpreted in the context of limitations associated with the data and methods. As with all claims databases, prescription fills do not account for whether the medication dispensed was taken as prescribed, which may have led to persistence on ustekinumab being overestimated. It was also not possible to draw conclusions regarding other clinical outcomes such as response to treatment, as the data source did not contain this information; a dataset in which claims data are linked to patient medical records could allow a more detailed analysis of the relationship between treatment persistence and effectiveness. Moreover, days of supply were imputed, assuming that time to the next claim is a proxy for the intended frequency of administration. Analyses of administrative claims depend on correct diagnosis, procedure, and drug codes. Given the open nature of the data source, drug utilization captured through medical or pharmacy claims may have been underestimated. More specifically, this may have led to an underestimation of the proportion of bio-experienced patients and of the adherence and persistence of ustekinumab. Regarding the pre-/post-analysis, events naturally occurring over time (ie, remitting and relapsing cycles of the disease, start of the COVID-19 pandemic) may have influenced the observed association of treatment with nonbiologic medication use. As the SHS database does not include information on medical services and prescriptions outside of its network of healthcare providers, the medical and pharmacy history of some patients may not have been fully captured. Finally, results may not be generalizable to patients without health insurance or with insurance other than commercial, as well as to patients with UC and other immune-mediated diseases.

## Conclusions

In this real-world study, most patients (>70%) with UC initiated on ustekinumab and reaching the maintenance phase remained persistent after 12 months of maintenance therapy. Moreover, nonbiologic medication use post-ustekinumab initiation, notably corticosteroid use, was significantly lower compared to the period pre-ustekinumab. Given the complications associated with chronic corticosteroid use, this reduction can be seen as clinically relevant and informs treatment choice for patients with UC. Further studies on real-world treatment patterns in patients with UC initiated on ustekinumab are needed and could notably investigate the impact of dose escalation on persistence.

## Data Availability

The data that support the findings of this study are available from Symphony Health (SHS), an ICON plc Company, PatientSource®, but restrictions apply to the availability of these data, which were used under license for the current study, and so are not publicly available. Any researchers interested in obtaining the data used in this study can access database through Symphony Health, under a license agreement, including the payment of appropriate license fee.

## Prior Presentation

Part of the results were presented at Digestive Disease Week 2022 (May 21–24, 2022, San Diego, CA) and Digestive Disease Week 2023 (May 6–9, 2023, Chicago, IL).
